# HLA-mismatched GPBSC infusion therapy in refractory Epstein-Barr virus-associated hemophagocytic lymphohistiocytosis: an observational study from a single center

**DOI:** 10.1186/s13287-020-01779-4

**Published:** 2020-07-01

**Authors:** Yue Song, Jingshi Wang, Yini Wang, Zhao Wang

**Affiliations:** Department of Hematology, Beijing Friendship Hospital, Capital Medical University, YongAn Road 95th Xicheng District, Beijing, 100050 China

**Keywords:** Hemophagocytic lymphohistiocytosis, Epstein-Barr virus, GPBSC infusion, Salvage therapy

## Abstract

**Background:**

Hemophagocytic lymphohistiocytosis (HLH) is a severe or even fatal inflammatory state. Epstein–Barr virus (EBV) infection-associated HLH (EBV-HLH) is one of the most common secondary HLH and suffers a very poor prognosis. Allo-HSCT is often required for refractory EBV-HLH, but some patients still cannot proceed to the next allo-HSCT due to various factors. This study aimed to observe the efficacy of HLA-mismatched granulocyte colony-stimulating factor (G-CSF)-mobilized peripheral blood stem cells (GPBSCs) infusion for refractory EBV-HLH.

**Methods:**

A retrospective case-control study of refractory EBV-HLH patients with GPBSC infusion from HLA-mismatched donors after chemotherapy (as GPBSC group) and sole chemotherapy (as control group) was performed. Efficacy was evaluated 2 and 4 weeks and all patients were followed-up until March 1, 2018.

**Results:**

There were 18 cases who accepted infusion between March 2016 and Sep 2017 and 19 were randomly selected from refractory EBV-HLH patients who underwent salvage therapy during the same period for the control group. In GPBSC group, WBC (*p* = 0.017), Fbg (*p* = 0.040), and ferritin (*p* = 0.039) improved significantly after treatment. The overall response rate was 66.7% (CR 22.2%, PR 44.4%). However, there are no significant differences in changes of WBC, HGB, PLT, TG, Fbg, Ferritin, AST, ALT, and T-bil between two groups. Only the Fbg level was recovered better in the GPBSC infusion group (*p* = 0.003). In the GPBSC group, EBV-DNA decreased significantly after 2 weeks (*p* = 0.001) and 4 weeks (*p* = 0.012) after treatment, and the effect of the decrease was significantly better than that of the chemotherapy alone group in 2 weeks but not 4 weeks (p2w = 0.011, p4w = 0.145). The median survival time in the infusion group was 20.4 weeks [95% CI 10.9, 29.9], and the median survival time in the control group was 10.8 weeks [95% CI 0–24.34]. In the short-term, the infusion group’s survival rate was better (2-month 88.89% vs. 52.63%, *p* = 0.008; 3-month 83.33% vs. 47.09%, *p* = 0.012), but there was no difference in OS (*p* = 0.287).

**Conclusions:**

Infusing GPBSCs combined with chemotherapy is effective, especially in decreasing EBV-DNA, performs better than chemotherapy alone, and improves short-term survival rate. GPBSC infusion is suggested as a bridging treatment method to allo-HSCT.

## Background

Hemophagocytic lymphohistiocytosis (HLH) is a severe or even fatal inflammatory state caused by a hereditary or acquired immunoregulatory abnormality, non-malignant proliferation of lymphocytes and tissue cells, and secretion of a large number of inflammatory cytokines [1]. HLH is divided into two categories: primary and acquired. Acquired HLH is often associated with and caused by infections, malignancies, and autoimmune diseases [2]. Among the infection-associated HLH, Epstein–Barr virus (EBV) infection-associated HLH (EBV-HLH) is one of the most common. In previous studies, EBV-HLH patients suffered a much worse prognosis than patients with other types of infection-associated HLH, especially in adult HLH patients [3–5]. In 2015, a study of 61 cases with EBV-HLH reported a 1-year overall survival (OS) of only 25.0% [6]. The current first-line treatment regimen, HLH-94 followed by allo-HSCT fails to trigger a response under ideal conditions in 30% of children with all HLH triggers [7]. In a study of 133 adults and adolescents with EBV-HLH, the non-response rate of the HLH-94 regimen was 52% [5]. The DEP (doxorubicin-etoposide-methylprednisolone) regimen and L-DEP (PEG-asparaginase and DEP regimen combination) regimen, which is used as a salvage therapy for refractory EBV-HLH, achieve a much better overall response rate and increase the chance to receive allo-HSCT, which improves survival [8–10]. However, some patients still cannot proceed to the next allo-HSCT due to various factors, such as financial limitations, disease activity, and a lack of time to find a suitable donor. It has been reported that granulocyte colony-stimulating factor (G-CSF)-mobilized peripheral blood stem cell (GPBSC) infusion can mediate graft-versus-leukemia (GVL) effects and hasten hematologic recovery without amplifying graft-vs-host disease (GVHD) [11]. Additionally, many studies have shown that infusion of HLA-mismatched donor GPBSCs combined with chemotherapy increased CR rates, improved survival, and avoided GVHD in AML patients [12–15]. In this study, patients with refractory EBV-HLH and the inability to undergo allo-HSCT were treated with GPBSC infusion after chemotherapy. What is the effect of this treatment method on HLH? Can it contribute to hematologic recovery, EBV-DNA reduction, and prognosis improvement? Is it possible that GPBSC infusion can be a bridge to allo-HSCT for these patients? These issues are discussed in this retrospective study.

## Methods

### Patients and donors

The study was approved by the Ethics Committee at Beijing Friendship Hospital. Written informed consent was obtained from each patient and/or their family or guardian before the treatment began.

A retrospective case-control study of refractory EBV-HLH patients with GPBSC infusion from HLA-mismatched donors after chemotherapy (GPBSC group) and sole chemotherapy (control group) was conducted. The patients in the control group were randomly selected from refractory EBV-HLH patients who underwent salvage therapy during the same period. Patients who were enrolled in this study fulfilled the following criteria: (1) patients satisfied HLH-2004 criteria at the time of initial diagnosis (the NK cell activity was tested by flow cytometry, ZL 201610013454.1) [1]; (2) high values for EBV-DNA copies in the peripheral blood (> 5.0 × 10^2^ copies/ml) (tested with PCR assay); (3) primary HLH was excluded by HLH-related defective gene proteins PRF, GrB, XIAP, SAP, Munc13-4, Munc18-2, STX11, Rab27a, ITK, CD27, AP3B1, and whole genome sequencing if necessary, and lymphoma was excluded by repeated pathological biopsy of the focal area if there was an abnormal increase in FDG activity tested by positron emission tomography-computed tomography (PET-CT) and bone marrow biopsy; (4) treated with HLH-94 regimen (etoposide 150 mg/m^2^ twice weekly for 2 weeks and then weekly) and dexamethasone (initially 10 mg/m^2^ for 2 weeks followed by 5 mg/m^2^ for 2 weeks, 2.5 mg/m^2^ for 2 weeks, 1.25 mg/m^2^ for 1 week, and 1 week of tapering) [7] no less than 2 weeks before enrolment and did not achieve at least PR; and (5) patients are unable to perform allo-HSCT at that time point due to lack of donors, financial, patient and/or families’ refusal, and hesitating or other reasons, such as economic limitation.

EBV infection was confirmed by identifying a significantly increased number of EBV-DNA copies in the peripheral blood. In the absence of the accepted diagnostic criteria, refractory HLH was defined according to previous research findings [16] and our clinical experience as failure to achieve at least PR according to an evaluation 2 weeks after receiving HLH-94 induction therapy.

Before GPBSC infusion, the HLA matching of donors and recipients was tested. The HLA matching of the GPBSC infusion group was HLA-mismatched with relative donors. All the donors were haplo-identical and at least 5/10.

### Treatment methods

Two kinds of treatment methods for patients: (1) GPBSC group: GPBSC infusion from HLA-mismatched donors after chemotherapy, in which the infusion of the cells was performed at 36 h after the chemotherapy regimen on day 0; (2) control group: treated only with chemotherapy. The chemotherapy regimen included salvage therapy: DEP regimen (liposomal doxorubicin 25 mg/m^2^ day 1; etoposide 100 mg/m^2^ was administered once on the first day of every week; methylprednisolone 15 mg/kg days 1 to 3, 2 mg/kg days 4 to 6, 1 mg/kg days 7 to 10, 0.75 mg/kg days 11 to 14, 0.5 mg/kg days 15 to 21, and 0.4 mg/kg days 22 to 28) and L-DEP regimen (PEG-asparaginase 2000 U/m^2^ on day 5; liposomal doxorubicin (doxorubicin hydrochloride liposome injection) 25 mg/m^2^/day, day 1; etoposide 100 mg/m^2^/day on the first day of every week; and methylprednisolone 15 mg/kg/day for days 1 to 3, 0.75 mg/kg/day for days 4 to 7, and 0.25 mg/kg/day for days 8 to 10).

### Mobilization and apheresis of donor peripheral mononuclear cells

Apheresis of HLA-mismatched donor peripheral mononuclear cells was carried out after the donor was subcutaneously injected with 5 μg/kg G-CSF twice a day for 5 days. Donor cells were divided into aliquots and were cryopreserved in liquid nitrogen, but freshly collected cells were used in the first course.

### Detection of donor chimerism and donor microchimerism

All patients in the GPBSC infusion group were assessed for donor chimerism after infusion. Eight patients were evaluated for donor microchimerism at least 1 week and 2 weeks after infusion.

### Evaluation criteria and observed indicators

Efficacy was evaluated 2 and 4 weeks after initiating therapy according to the criteria proposed by Marsh et al. [16]. Complete response (CR) was defined as the normalization of all quantifiable symptoms and laboratory markers of HLH, including the levels of soluble CD25, ferritin, and triglyceride; hemoglobin levels; neutrophil and platelet counts; and alanine aminotransferase (ALT) levels. Partial response (PR) was defined as improvement in two or more of the following quantifiable symptoms and laboratory markers by 2 weeks: 1.5-fold decrease in soluble CD25 response; ferritin and triglyceride decreases of at least 25%; an increase of at least 100% to > 0.5 × 10^9^/L in patients with an initial neutrophil count of < 0.5 × 10^9^/L; an increase by at least 100% to > 2.0 × 10^9^/L in patients with an initial neutrophil count of 0.5 to 2.0 × 10^9^/L; and a decrease of at least 50% in patients with initial ALT levels > 400 U/L. Additionally, the subject’s body temperature had to have reverted to normal ranges for either CR or PR to be diagnosed. Failure to achieve PR was defined as no response.

The observational indicators included symptoms and laboratory findings as indicated in the evaluation criteria. EBV-DNA copies in the peripheral blood were also observed.

### Survival time

Survival times were calculated from the date of diagnosis of refractory EBV-HLH. All patients were followed-up until death or March 1, 2018, whichever occurred first.

### Statistical analysis

SPSS 22.0 (IBM, USA) statistical software was adopted. Data that fit a normal distribution are presented as average ± standard deviation, and those that did not are presented as median and range. *T* test (two-sided) was used for data that fit a normal distribution and homogeneity of variance, and the Wilcoxon rank-sum test was used for others. Kaplan–Meier survival curves were used to analyze the patients’ survival, and the log-rank test was used to evaluate survival time. *P* < 0.05 was considered to denote a significant difference. Sample size calculation was performed using PASS 15 Power Analysis and Sample Size Software (2017, NCSS, LLC. Kaysville, Utah, USA) with *β* = 0.1 and *α* = 0.05 (two independent proportions).

## Results

### General conditions

There were 18 cases of refractory EBV-HLH who accepted GPBSC infusion between March 2016 and Sep 2017 as the infusion group. The reasons these patients did not receive allo-HSCT at that time point were lack of donors (*n* = 3), financial considerations (*n* = 3), and patient and/or families’ refusal (*n* = 5). The other 7 patients were hesitant about HSCT at that time point. In total, 19 patients were randomly selected from refractory EBV-HLH patients who underwent salvage therapy during the same period as the control group. The characteristics of the patients in the 2 groups are summarized in Table 1.

**Table 1:** Clinical features of patients in two groups before treatment

Clinical features	No. of patients		*p* valve
	Infusion group (*n* = 18)	Control group (*n* = 19)	
Median age, years	21 [11–60]	29 [17–57]	0.368
Gender, female to male	5:13	6:13	0.800
Fever	18 (100%)	19 (100%)	–
Splenomegaly/hepatomegaly	16 (88.9%)	16 (84.2%)	0.677
Hemophagocytosis	14 (77.8%)	14 (73.7%)	0.772
NK cell activity < 15.11%	6 (33.3%)	4 (21.1%)	0.401
soluble CD25 > 6400 pg/ml	14 (77.8%)	19 (100%)	0.100
EBV-DNA (copies/ml) (at diagnosis)	5.0 × 10^5^ [1.5 × 10^3^, 7.7 × 10^7^]	1.0 × 10^6^ [2.0 × 10^3^, 5.2 × 10^7^]	0.090

### Laboratory findings

There was no difference in HLH features before treatment in the GPBSC infusion group and the control group (including WBC, HGB, PLT, TG, Fbg, Ferritin, AST, ALT, T-bil) (*p* > 0.05). After 2 weeks of treatment in the GPBSC group, some of the patients’ laboratory indicators improved significantly, including WBC (*p* = 0.017), Fbg (*p* = 0.040), and ferritin (*p* = 0.039). The overall response rate was 66.7% (12/18), with a CR rate of 22.2% (4/18) and a PR rate of 44.4% (8/18). However, there were no significant differences in WBC, HGB, PLT, TG, Fbg, Ferritin, AST, ALT, and T-bil between the two groups. Only the Fbg level was recovered better in the GPBSC infusion group (*p* = 0.003), indicating that the GPBSC group was similar to the chemotherapy alone group in terms of hematological recovery. The details are presented in Table 2.

**Table 2:** Comparison between two treating groups

	Before		*p* value	2 weeks		*p* value
	GPBSCs	control		GPBSCs	control	
WBC	1.36 [0.2, 2.4]	1.8 [0.5, 7.4]	0.134	2.68 [0.17, 15.38]	3.95 [0.2, 12]	0.696
HGB	81.5 ± 17.701	94.63 ± 27.132	0.092	82.75 ± 18.614	97.19 ± 17.608	0.032
PLT	49.5 [12, 340]	45 [3, 499]	0.893	47 [3, 313]	88 [26, 451]	0.102
TG	2.59 [0.6, 12.78]	2.05 [1.39, 5.29]	0.929	1.89 [1.02, 8.86]	1.63 [0.74, 4.11]	0.093
Fbg	1.04 [0.84, 2.91]	1.045 [0.3, 2.12]	0.252	2.16 [0.77, 4.24]	1.065 [0.53, 2.74]	0.003
Ferritin	3843.1 [28, 24,174]	3207 [118, 455,000]	0.909	3352.5 [255, 40,699]	1433 [16, 7549]	0.068
AST	52.5 [13.3, 229.9]	65 [26, 766]	0.150	35.7 [12.8, 181]	28 [8, 680.2]	0.261
ALT	58 [14, 252]	76 [10, 310]	0.425	59 [6, 502]	34.5 [4, 227]	0.123
T-bil	18.725 [8.01, 85.66]	50.925 [7.48, 289.66]	0.088	33.51 [9.36, 169.14]	17.34 [1.84, 192.69]	0.551
EBV-DNA	1.1 × 10^5^ [< 5.0 × 10^2^, 3.0 × 10^6^]	5.7 × 10^5^ [< 5.0 × 10^2^, 5.2 × 10^7^]	0.304	5.0 × 10^3^ [< 5.0 × 10^2^, 5.7 × 10^5^]	6.6 × 10^4^ [< 5.0 × 10^2^, 9.0 × 10^7^]	0.011
ECOG	2.78	2.63	0.606	2.56	2.42	0.645

**Fig. 1:**
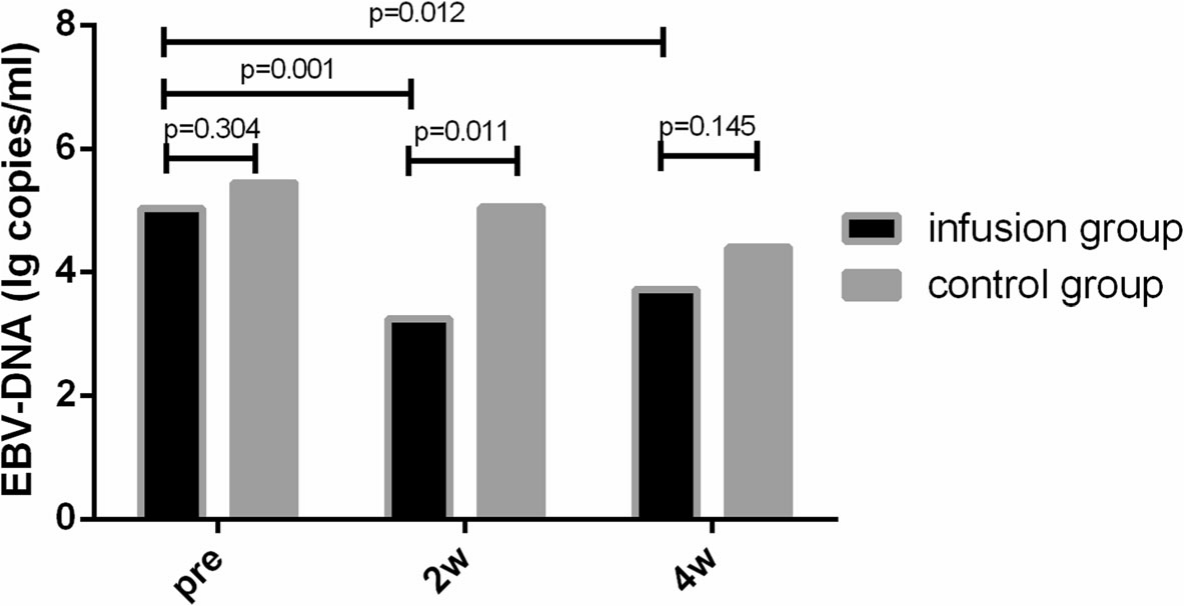
EBV-DNA before, 2 weeks and 4 weeks after treatment between two groups. There was no difference in EBV-DNA levels before treatment (*p* = 0.304) between two groups. In the GPBSC group, EBV-DNA decreased significantly after 2 weeks (*p* = 0.001) and 4 weeks (*p* = 0.012) after treatment, and the effect of the decrease was significantly better than that of the chemotherapy alone group in 2 weeks but not 4 weeks (p2w = 0.011, p4w = 0.145)

In terms of EBV, there was no difference in EBV-DNA levels at diagnosis (5.0 × 10^5^ [1.5 × 10^3^, 7.7 × 10^7^] vs. 1.0 × 10^6^ [2.0 × 10^3^, 5.2 × 10^7^], *p* = 0.090) or before treatment (1.1 × 10^5^ [< 5.0 × 10^2^, 3.0 × 10^6^] vs. 5.7 × 10^5^ [< 5.0 × 10^2^, 5.2 × 10^7^], *p* = 0.951) between the two groups. In the GPBSC group, EBV-DNA decreased significantly after 2 weeks (5.0 × 10^3^ [< 5.0 × 10^2^, 5.7 × 10^5^], *p* = 0.001) and 4 weeks (4.1 × 10^3^ [0, 4.4 × 10^4^], *p* = 0.012) after treatment, and the effect of the decrease was significantly better than that of the chemotherapy alone group in 2 weeks but not 4 weeks (p_2w_ = 0.011, p_4w_ = 0.145) (Fig. 1).

### Survival analysis

**Fig. 2:**
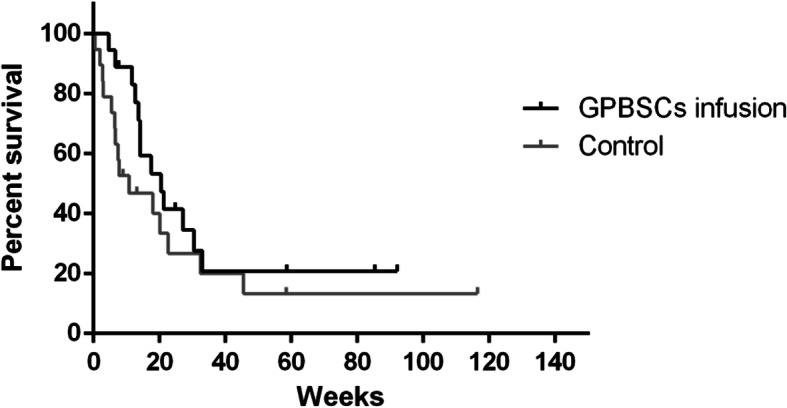
Overall survival (OS) between two groups (*p* = 0.259)

The statistical analysis of the survival times ended on March 1, 2018. The overall mortality of the 34 patients was 75.7% (28/37), and the median survival time was 14.14 weeks [0.6–116.4]. A total of 13 of 18 in the GPBSC infusion group died (72.2%); 7 of them died of HLH progression, 1 died of aGVHD after GPBSC infusion, 4 died of allo-HSCT-related causes, and 1 died of complications. The median survival time of the infusion group was 20.4 weeks [95% CI 10.9, 29.9]. A total of 15 of 19 in the control group died (78.9%); 8 of them died of HLH progression, 3 died of allo-HSCT-related causes, and 4 died of complications. The median survival time of the control group was 10.8 weeks [95% CI 0–24.34–116.4]. In the first 3 months after treatment, the GPBSC infusion group’s survival was better than that of the control group (2-month survival 88.89% vs. 52.63%, *p* = 0.008; 3-month survival 83.33% vs. 47.09%, *p* = 0.012). However, after 3 months, there was no difference between the two groups. In addition, there was no significant difference in overall survival (OS) between the two groups (*p* = 0.287) (Fig. 2).

### Graft-versus-host disease (GVHD)

The median number of mononuclear cells (MNCs) infused was 7.60 × 10^8^/kg (range, 5.1–12.2 × 10^8^/kg). The median infused CD34+ cell number was 2.355 × 10^6^/kg (range, 1.17–4.924 × 10^6^/kg). The number of infused cells was mainly to ensure that the infusion was effective. At present, there are no standard recommendations on the doses of infused cells for GPBSC infusion therapy. The doses of CD34 + cells infused in this study were with reference to the doses of cells (CD34+ 1.7 × 10^6^/kg, range, 1.1–4.6) × 10^6^/kg) infused in the study of Mei Guo et al. [13]. Among the 18 patients who underwent GPBSC infusion, 7 (38.9%) had acute GVHD (aGVHD) signs, 5 of which had liver dysfunction and gastrointestinal symptoms, and the other 2 cases had liver dysfunction or gastrointestinal symptoms. However, 6 of them improved after symptom-specific treatment. Only 1 patient experienced sustained and eventually died of aGVHD. The details of graft and GVHD are presented in Table 3.

**Table 3:** Graft details and donor microchimerism rate after GPBSC infusion

Patient	Graft		Donor microchimerism rate (%)				aGVHD	
	Mononuclear (cells/kg)	CD34+ (cells/kg)	1 week	2 weeks	3 weeks	4 weeks	Organ system affected	Severity scoring
1	12.2	4.56	37.4	86.0	93.89		Elevated LFTs, rash, GI symptoms	IV
2	5.5	2.31	0.024	0.081				
3	9.7	2.115	0.023	0.014	0.005			
4	5.5	2.59	–					
5	5.1	1.17	0.846	0.148			Elevated LFTs, GI symptoms	II
6	5.2	2.08	0.17				Elevated LFTs, GI symptoms	II
7	6.4	1.4	–					
8	11.6	2.43	0.15	0.25				
9	10.1	2.4	–				GI symptoms	I
10	9.7	4.924	11.21	0.15	0.01	0.01	Elevated LFTs, GI symptoms	II
11	16.4	3.19	–					
12	5.3	1.325	0.002	0.001			Elevated LFTs	I
13	9.6	3.11	0.008					
14	6.1	2.04	–					
15	8.8	2.65	–					
16	16.5	2.31	0.37	0.31	0.034			
17	5.1	2.448	–					
18	5.7	1.824	3.26	0.76			Elevated LFTs, GI	II

*LFT* liver function test, *GI* gastrointestinal

### Donor chimerism and donor microchimerism

Only one patient in the GPBSC group developed mixed chimerism, and the highest peripheral chimerism rate was 93.45% (3 weeks after treatment). This patient eventually died of aGVHD. In the other 10 patients who underwent microchimerism detection, micro-chimerism was detected (0.008–3.26%). In 2 weeks after treatment, microchimerism was detected in 8/8. There were 3 patients whose microchimerism was also detected again at 3 weeks (Table 3).

## Discussion

EBV-HLH suffers a poorer prognosis than other subtypes of secondary HLH, especially relapsed and refractory EBV-HLH [17, 18]. Without effective treatment, short-term mortality is high, and most patients with relapsed EBH-HLH die within the first few weeks [7]. Allogeneic hematopoietic stem cell transplantation (allo-HSCT) is often required for EBV-HLH, especially for adults and refractory/relapsed patients. It is thought that allo-HSCT can induce immune reconstitution, thus enabling patients to effectively eliminate EB virus [3, 19]. In one Japanese cohort, allo-HSCT resulted in an 85.7% 10-year OS for patients with EBV-HLH [20]. In a retrospective analysis from our center, 27.1% of patients with EBV-HLH received allo-HSCT, and the final survival rate was 52.78% [5]. However, some patients in the actual situation are unable to receive allo-HCST due to various factors. It has been reported that granulocyte colony-stimulating factor (G-CSF)-mobilized peripheral blood stem cell (GPBSCs) infusion can mediate GVL effects and hasten hematologic recovery without amplifying GVHD in AML [11]. Considering the important position of allo-HSCT, we tried to use GPBSC infusion as a salvage treatment in patients with refractory EBV-HLH. This study found that for patients with refractory EBV-HLH, GPBSC infusion therapy could more effectively reduce EBV-DNA levels after 2 and 4 weeks but had no significant effect on the recovery of blood parameters. The 3-month survival was improved, but there was no significant effect on overall survival.

The G\PBSC infusion therapy in the treatment of leukemia reported in previous literature can significantly shorten the recovery time of white blood cells and platelets [11]. GPBSCs is a stimulated cell infusion. The current speculation is that, in contrast to unstimulated DLI, the larger numbers of lymphocytes, CD34 cells, natural killer (NK) cells, and cytokines, contained in G-PBSCs, may contribute roles in promoting hematopoietic recovery [13, 21]. However, in this study, there was no significant difference in the recovery of blood parameters in the GPBSC infusion group compared with the uninfused group, although similar levels of donor chimerism were observed in this study, which was consistent with the previous study of leukemia. It is possible that the disease state of HLH is different from that of leukemia. The decline in blood cells in HLH is mainly related to the production and action of a large number of inflammatory mediators [19]. This is different from the bone marrow suppression caused by chemotherapy drugs in the treatment of leukemia. HLH needs to be controlled by controlling inflammatory cells and then controlling the inflammatory factor storm [22]. However, the mechanism of hematopoietic recovery of GPBSCs is still unclear, especially in the context HLH, and the specific internal mechanism still needs further study.

Interestingly, GPBSC infusion after chemotherapy was more effective at reducing EBV-DNA viral load than regular chemotherapy. This may be similar to the GVL effect of GPBSCs in the treatment of leukemia: the lymphocytes of the infused donor cells act synergistically with the recipient’s immune system to delete EBV-infected cells, thereby reducing Epstein-Barr virus load. However, prior studies suggested that the main mediator of GVL in leukemia is NK cells, and other cells, such as T cells, mainly function in GVL by interacting with the recipient’s immune system [13, 23]. However, in HLH, EB virus clearance mainly relies on EB virus-specific CD8+ T cells [24]. Previous studies have found that EBV-specific CD8+ T recombination occurs in the early phase of allo-PBSCT; even if the total number of T cells decreased, the proportion of CD8+ T is still elevated, but this phenomenon was not observed in cord blood transplantation [25]. The GVL effect of DLI is mainly through donor chimerism or mixed chimerism [23]. In this study, only one patient achieved mixed chimerism after the infusion of cells, although the number of cells infused was not different from those of other patients. This patient eventually died of GVHD, but the EBV viral load of this patient decreased significantly, and EBV-DNA turned negative after 4 weeks, suggesting that the effect of the reduction of EBV-DNA is achieved by the GVL-like effect of lymphocytes in the infused cells, similar to that observed in leukemia. With the patients who were tested for chimerism in this study, all of them had microchimerism except the patient mentioned above. Almost all patients with microchimerism did not develop mixed chimerism, suggesting that donor or mixed chimerism is difficult to achieve with GPBSC infusion. However, with the persistence of micro-chimerism, a small number of donor cells persist in the patient; this micro-chimerism may be the main reason by which GPBSCs effectively reduce the EBV-DNA level. Due to the small sample size of this study, no correlation between the micro-chimerism rate and the decline in EBV-DNA levels was found. However, in the previous micro-transplant study, the micro-chimerism rate reached a peak at 7–14 days after infusion [11]. This is consistent with the efficacy of EBV-DNA’s reduction, which was maximal in 2 weeks, but more data are needed to validate the relationship. However, the efficacy of EBV-DNA reduction cannot persist; as in 4 weeks after treatment, there is no difference in EBV-DNA level between the 2 groups. We speculate that the loss of differences in EBV viral load is related to possible depletion of GPBSCs. Considering the transient efficacy of GPBSC infusion, allo-HSCT should be taken into consideration as soon as HLH is controlled. Therefore, GPBSC infusion may play a role in bridging the allo-HSCT, especially for those patients who have not had a previous transplant opportunity.

Except for the one patient mentioned above, who achieved mixed chimerism and suffered severe GVHD causing death, only 6 of the remaining 14 patients developed aGVHD signs, and all of them improved rapidly after symptomatic treatment, even if the number of CD3+ cells infused was high. None of the 15 patients in the GPBSC infusion group underwent GVHD prevention treatment. Regardless of DLI or haploid transplantation, although the GVL effect is very impressive, the GVHD is also very severe [26]. Although GPBSC infusion is a kind of transplantation with infused donor cells and chemotherapy, it was not used as a pretreatment regimen with strong immunosuppressive effects similar to haploid transplantation [11]. No severe immunosuppression significantly reduces the incidence and severity of GVHD after infusion. However, considering that one patient died of severe GVHD in this study, more considerations are required for special circumstances, and close monitoring of GVHD after the infusion is still necessary.

In terms of survival, the survival rate of the GPBSC infusion group was better than that of the control group within 3 months, but there was no significant difference in the long-term survival. This may suggest that GPBSC infusion can help with refractory EBV-HLH, but only provides effects in the short term. Allo-HSCT is still needed to achieve long-term remission [27]. Therefore, we suggest using GPBSCs as a bridging treatment method to allo-HSCT for those refractory EBV-HLH patients who did not have a previous opportunity for allo-HSCT.

## Conclusion

Infusing HLA-mismatched donor GPBSCs combined with chemotherapy is effective in refractory EBV-HLH. It can decrease EBV-DNA levels in 2 and 4 weeks, and it performs better than chemotherapy alone in 2 weeks. Infusing HLA-mismatched donor GPBSCs combined with chemotherapy can also improve the short-term survival rate. This effect is similar to the effect of GVL in leukemia, but it is not exactly the same, which is probably due to the GVL-like effect of lymphocytes in the infused cells, and microchimerism may be important. However, the effects are short-term. After the exhaustion of donor cells, allo-HSCT is still needed. GPBSC infusion is suggested as a bridging treatment method to allo-HSCT for those refractory EBV-HLH patients who have not had a previous opportunity for transplantation.

## Data Availability

The datasets used during the current study are available from the corresponding author on request.

## Abbreviations

Allo-HSCT: Allogeneic hematopoietic stem cell transplantation
ALT: Alanine aminotransferase
CR: Complete response
DEP regimen: Doxorubicin hydrochloride liposome, etoposide, and methylprednisolone
EBER: EBV-encoded small RNA
EBV: Epstein–Barr virus
GVHD: Graft versus host disease
HLH: Hemophagocytic lymphohistiocytosis
L-DEP regimen: PEG-aspargase plus DEP regimen
NK: Natural killer
OS: Overall survival
PR: Partial response
VP-16: Etoposide
GPBSCs: Granulocyte colony-stimulating factor-mobilized peripheral blood stem cells
